# The RNA-binding protein PCBP1 represses lung adenocarcinoma progression by stabilizing DKK1 mRNA and subsequently downregulating β-catenin

**DOI:** 10.1186/s12967-022-03552-y

**Published:** 2022-07-30

**Authors:** Yujia Zheng, Zheng Zhou, Ran Wei, Chu Xiao, Hao Zhang, Tao Fan, Bo Zheng, Chunxiang Li, Jie He

**Affiliations:** 1 Department of Thoracic Surgery, National Cancer Center/National Clinical Research Center for Cancer/Cancer Hospital, Chinese Academy of Medical Sciences and Peking Union Medical College, Beijing, China; 2 Department of Colorectal Surgery, National Cancer Center/National Clinical Research Center for Cancer/Cancer Hospital, Chinese Academy of Medical Sciences and Peking Union Medical College, Beijing, China; 3 Department of Pathology, National Cancer Center/National Clinical Research Center for Cancer/Cancer Hospital, Chinese Academy of Medical Sciences and Peking Union Medical College, Beijing, China

**Keywords:** PCBP1, DKK1, LUAD, Wnt signalling pathway

## Abstract

**Background:**

PolyC-RNA-binding protein 1 (PCBP1) functions as a tumour suppressor and RNA regulator that is downregulated in human cancers. Here, we aimed to reveal the biological function of PCBP1 in lung adenocarcinoma (LUAD).

**Methods:**

First, PCBP1 was identified as an important biomarker that maintains LUAD through The Cancer Genome Atlas (TCGA) project screening and confirmed by immunohistochemistry and qPCR. Via colony formation, CCK8, IncuCyte cell proliferation, wound healing and Transwell assays, we confirmed that PCBP1 was closely related to the proliferation and migration of LUAD cells. The downstream gene DKK1 was discovered by RNA sequencing of PCBP1 knockdown cells. The underlying mechanisms were further investigated using western blot, qPCR, RIP, RNA pulldown and mRNA stability assays.

**Results:**

We demonstrate that PCBP1 is downregulated in LUAD tumour tissues. The reduction in PCBP1 promotes the proliferation, migration and invasion of LUAD in vitro and in vivo. Mechanistically, the RNA-binding protein PCBP1 represses LUAD by stabilizing *DKK1* mRNA. Subsequently, decreased expression of the DKK1 protein relieves the inhibitory effect on the Wnt/β-catenin signalling pathway. Taken together, these results show that PCBP1 acts as a tumour suppressor gene, inhibiting the tumorigenesis of LUAD.

**Conclusions:**

We found that PCBP1 inhibits LUAD development by upregulating DKK1 to inactivate the Wnt/β-catenin pathway. Our findings highlight the potential of PCBP1 as a promising therapeutic target.

**Supplementary Information:**

The online version contains supplementary material available at 10.1186/s12967-022-03552-y.

## Introduction

Lung cancer is the second most common diagnosed malignancy and the leading cause of cancer death worldwide [1]. Lung adenocarcinoma (LUAD) is the most common histological subtype of lung cancer [2], and the 5-year survival rate is less than 30% [3]. Metastatic disease is a poor prognostic feature and a leading cause of death in LUAD patients [4, 5]. Rapid disease progression and organ failure due to metastasis account for the majority of patient mortality [6, 7]. Metastatic lung adenocarcinoma cells can spread through the blood and lymphatic circulation, eventually colonizing the liver, bone, brain, and other organs. Unfortunately, despite significant advances that have been achieved in understanding the precise molecular mechanisms involved in LUAD, its mechanisms of development, progression, and metastasis remain elusive.

PCBPs are a subgroup belonging to the heterogeneous nuclear ribonuclear protein (hnRNP) family and contain three conserved RNA-binding domains, termed the heterogeneous nuclear protein k-homology (KH) domain, which has specific roles in interactions with RNAs and proteins, thereby regulating gene expression at various level [8]. PolyC-RNA-binding protein 1 (PCBP1) is also known as hnRNP E1 [9], which was identified as a component of heterogeneous nuclear ribonucleoprotein complexes and regulates alternative splicing, translation, and RNA stability of many cancer-related genes [10]. PCBP1 can reduce the stability of LC3B and p62/SQSTM1 mRNA [11, 12]. PCBP1 also plays a role in CD44 alternative splicing [13]. PCBP1 regulates gene expression by binding to specific elements of its target mRNAs (e.g., p21, p63, and c-myc) [14–16].

PCBP1 has been shown to be a tumour suppressor and is closely related to the occurrence and development of various tumours. In haematological tumours, PCBP1 was found to be a more common mutation type after the common mutations in Burkitt lymphoma with respect to ID3, TCF3, CCND3, and TP53 [17]. Large-scale sequencing results revealed that mutations in PCBP1 also frequently occur in colon cancer [18]. In prostate cancer, TGF-β1 enhances tumour stemness by downregulating expression of PCBP1 [19]. In ovarian cancer, PCBP1 inhibits tumour progression by regulating the mRNA stability of p27 [20]. In breast cancer, knockdown of PCBP1 enhances the stemness of breast cancer cells and promotes breast cancer invasion and metastasis by regulating the ILEI/LIFR signalling pathway [21]. In gastric and pancreatic cancer, downregulation of PCBP1 expression promotes peritoneal metastasis [22–24]. In recent years, researchers have attempted to elucidate the multiple regulatory roles of PCBP1 in tumorigenesis, development and metastasis; however, the function of PCBP1 in LUAD tumorigenesis and metastasis remains unknown.

Cancer metastasis, a process involving the spread of cancer cells from a primary lesion to distant organs, is one of the greatest contributors to cancer-related death [25]. The Wnt signalling pathway and its involvement in cancers have been extensively investigated and play an important role in the development and metastasis of cancer [26, 27]. This signalling pathway affects the maintenance of metastasis in lung cancer and provides targets for developing cancer therapy agents [28]. Since the reduction of PCBP1 is a key alteration contributing to the acquisition of metastatic characteristics in tumour cells, understanding the function of PCBP1 is critical for its development as a therapeutic target to slow cancer progression. Utilizing functional studies, mechanistic investigations and mouse models, we demonstrate that PCBP1 inhibits LUAD development by upregulating DKK1 to inactivate the Wnt/β-catenin pathway. Our study highlights the important function of PCBP1 in LUAD, indicating its promising clinical potential as a prognostic marker and novel therapeutic target.

## Materials and methods

### Preprocessing of clinical and sequencing data and the heterogeneous nuclear ribonucleoprotein (hnRNP) family

This study incorporated data from two publicly available databases, the Cancer Genome Atlas (TCGA) and the Gene Expression Omnibus (GEO). LUAD samples from TCGA and normal tissue from Genotype-Tissue Expression (GTEx) were acquired from the UCSC Xena (https://tcga.xenahubs.net). Additional LUAD cases (n = 82) were selected from GSE19188. Patients with an expression value of zero detected by more than 50% probes or missing prognostic data were excluded, while others were not included due to incomplete clinicopathological data. More gene expression and prognosis data of breast cancer, ovarian cancer, and gastric cancer patients were downloaded from the Kaplan–Meier (K-M) plotter database [29, 30]. A list of the hnRNP family was extracted from previously published studies [31].

### Differentially expressed, survival, and functional analysis

LUAD patients from TCGA were divided into an early death group (death within one year) and a long-term survival group (more than 5-year overall survival). Propensity score (PS) matching analysis was performed between the two groups to adjust for clinical features. The linear model for microarray data (LIMMA) method was used to evaluate differentially expressed genes (DEGs) between cancer and adjacent tissues in LUAD patients and those who had different prognoses. Kaplan–Meier survival curve analyses were performed on TCGA and GSE19188 datasets using the R package “survcomp” to determine the prognostic value of genetic markers [32]. Based on the "ggpubr" R package, the research team constructed box plots to show the specific gene expression levels of different samples. The correlations between gene expression and molecular or immune subtypes across cancers were explored using the TISIDB database [33]. Meanwhile, the research group performed Gene Set Enrichment Analysis (GSEA) using “javaGSEA” [34].

### Human samples

We obtained fresh tumour tissues and paired normal tissues from 31 patients and paraffin-embedded tissue samples from 72 patients with pathologically confirmed LUAD (Additional file 1: Table S1) who underwent surgery at the National Cancer Centre/National Clinical Research Centre for Cancer/Cancer Hospital, Chinese Academy of Medical Sciences and Peking Union Medical College (Beijing, China).

Clinical data were collected by reviewing the patients’ medical histories. Pathological staging was performed according to the 8th edition of the American Joint Committee on Cancer/Union for International Cancer Control TNM classification system.

### Cell culture

The human lung adenocarcinoma A549 and H358 cell lines were cultured in RPMI-1640 (Corning) supplemented with 10% foetal bovine serum (FBS, Gibco) and 100 U/mL penicillin–streptomycin (Gibco). Cells were cultured at 37 °C in a humidified atmosphere containing 5% CO_2_.

### Small interfering RNA (siRNA) and plasmid transfection

To establish stably interfered or target gene-expressing cells, we transfected lentivirus-mediated shRNA specifically against PCBP1 and lentiviral PCBP1 overexpression constructs into A549 and H358 cells according to the manufacturer’s protocols. Positively infected cells were selected using puromycin treatment for 3 weeks. Transfection of cells with siDKK1 was performed using Lipofectamine™ 3000 (Invitrogen, Carlsbad, USA) according to the manufacturer’s instructions.

### RNA isolation, reverse transcription, and qPCR

Total RNA was isolated from tissues or cells using RNAiso Plus (9108, TakaRa), and then total RNA was used for complementary DNA (cDNA) synthesis using the Prime Script RT reagent kit according to the manufacturer’s instructions (RR036A, TaKaRa). Relative mRNA expression levels were determined by qPCR using SYBR Green Master Mix (RR82LR, TaKaRa). β-actin and GAPDH served as internal controls, and the 2^−ΔΔCT^ method was utilized to calculate relative expression levels. Detailed primer sequences are listed in Additional file 1: Table S2.

### Western blot

Proteins were extracted from cells and animal tissues. Next, the proteins were transferred onto polyvinylidene fluoride membranes (Millipore, Bedford, MA) using electrophoresis and transferred to a 0.2-µm nitrocellulose membrane (GE Healthcare, Chicago, IL USA). Membranes were blocked with 5% nonfat milk and incubated with primary antibodies overnight at 4 ℃. The primary antibodies used for western blotting were as follows: PCBP1 (1:2000, ab74793, Abcam), DKK1 (1:1000, ab109416, Abcam), phosphor-β-catenin (1:1000, 9561S, CST), Claudin-1 (1:1000, 13255 s, CST), β-catenin (1:1000, 8480 s, CST), and Vimentin (1:1000, 5741 s, CST). The signalling detection was performed using an ECL detection kit (WBKLS0500, Millipore).

### mRNA stability assay

A549 cells stably expressing PCBP1 and control cells were treated with 2 mg/ml actinomycin D (SBR00013, Sigma) to inhibit mRNA transcription. Total RNA was extracted for cDNA synthesis and detected by semiquantitative and real-time RT–PCR as indicated. Relative mRNA levels were normalized to the starting point of treatment.

### IncuCyte™ cell proliferation and wound healing assays

IncuCyte live-cell imaging enables noninvasive, fully kinetic measurements of cell growth based on area (confluence) metrics. Cell cultures were imaged every twelve hours using IncuCyte (Essen BioScience, USA). A total of 3 × 10^3^ cells were seeded into 96-well plates, and cell proliferation was evaluated by the degree of cell confluence. A total of 8 × 10^4^ cells were plated in duplicate in 96-well plates and grown to 90% confluence. Wounds were then created using IncuCyte ZOOM™. After washes to remove cellular debris with PBS, serum-free medium was added. Plates were then cultured in an IncuCyte ZOOM™ incubator. The results of the cell proliferation and migration assays were analysed 72 h later.

### RNA sequencing (RNA-seq) and bioinformatics analyses

RNA-seq assays were performed at Novogene (China) to measure the mRNA expression profiles of A549 cells and shPCBP1 A549 cells using Illumina PE150. Differentially expressed genes (DEGs, |logFC|≥ 0.5) were clustered and visualized using the pheatmap R package. Gene Ontology (GO) and Kyoto Encyclopedia of Genes and Genomes (KEGG) pathway analyses of DEGs were performed using the clusterProfiler R package.

### RNA pulled down and silver staining

The RNA–Protein Pull-Down Kit (21,115, Thermo Fisher Scientific) was used according to the manufacturer’s instructions for RNA pulldown. Biotin-labelled DKK1 RNA and control RNA were obtained in vitro using the MEGAscript™ T7 Transcription Kit (AM1334, Invitrogen) and bio16-UTP (AM8452, Invitrogen). Biotinylated DKK1 RNA was captured using streptavidin magnetic beads and subsequently mixed with the A549 cell protein lysates. The compounds were separated by SDS–PAGE and subjected to western blotting and silver staining.

### RNA immunoprecipitation (RIP) assay

The EZ-Magna RIP Kit (17–701, Millipore) was used according to the manufacturer’s instructions. Immunoprecipitated RNA was extracted from the eluent and reverse transcribed. Protein–RNA complexes immunoprecipitated by anti-PCBP1 or IgG were determined through qPCR.

### Transwell assay

A transwell plate with an 8 μm porous polycarbonate membrane was used to assess tumour cell migration (3422, Corning). The tumour cells were suspended in serum-free RPMI 1640 and placed on the upper portion of a transwell chamber. The 10% FBS RPMI1640 placed in the lower portion of the chamber was used as a chemoattractant. After 24 h of incubation, the cells on the upper side were removed with cotton swabs, whereas the migrating cells on the lower side were fixed in 4% paraformaldehyde at room temperature for 30 min and then stained with 0.1% crystal violet for 30 min. Finally, the migrating cells were imaged and counted using ImageJ software.

### Immunohistochemistry (IHC)

The tissue slices were harvested and then processed with formalin, dehydrated, paraffinized and sectioned. Immunohistochemical staining was performed using antibodies against PCBP1 (1:200, ab109577, Abcam), DKK1 (1:200, ab109416, Abcam), β-catenin (1:200, 8480, CST) and Ki67 (1:200, ab 15,580, Abcam). The immunopathology score was assessed by two pathologists, and the total result was given according to the positive intensity and area extent. Each specimen received a score according to staining intensity (negative = 0, weak = 1, moderate = 2, and strong = 3) and the percentage of positive cells (0% = 0, 0–1% = 1, 2–10% = 2, 11–30% = 3, 31–70% = 4, and 71–100% = 5).

### CCK-8 assay

Transfected A549 and H358 cells were seeded into 96-well plates (2000 cells/well). Cell viability was assessed using the Cell Counting Kit-8 (CCK-8) assay (CK04, Dojindo) according to the manufacturer’s instructions. The absorbance was measured at 450 nm using a microplate reader (Thermo Fisher Scientific, Singapore).

### Colony formation assay

A549 and H358 cells were seeded into 6-well plates with 200 cells per well supplemented with 2 mL of 10% FBS cell culture medium, and the medium was changed every 3 days. After 10–14 days, harvested cells were fixed in 4% PFA for 30 min and stained with crystal violet. Three images of each well were acquired.

### Mouse models

Five-week-old female BALB/c nu mice were used to establish a subcutaneous xenograft model. A549 and H358 cells transfected with sh-PCBP1 or control and PCBP1 or NC were resuspended in PBS. Transfected A549 and H358 cells were inoculated subcutaneously in 100 μL PBS at a density of 1 × 10^6^ cells per mouse, with 6 mice in each group. Starting 12 days after inoculation, the tumours were measured every 3 days. The tumour volume (mm^3^) was assessed as follows: volume = (length × width^2^)/2.

Five-week-old female NSG mice were used to establish a metastatic lung model. In the metastatic lung model, 1 × 10^6^ cells were injected into the tail vein. After inoculation, mouse weight was measured every week. Eight weeks after the H358 injection or sixteen weeks after the A549 injection, mice were sacrificed to collect the lungs. Metastatic nodules were assessed using HE staining. This study was approved by the Institutional Animal Care and Use Committee of Peking Union Medical College, Chinese Academy of Medical Sciences.

### Statistical analysis

Statistical analyses were performed using Prism 7.0 or R. The paired Student's t test, chi-square test, Kaplan–Meier method and Spearman's correlation analyses were used (*P < 0.05; **P < 0.01). The data are reported as the mean ± S.D.

## Results

### Low expression of PCBP1 predicts poor prognosis in tumour patients

**Fig. 1:**
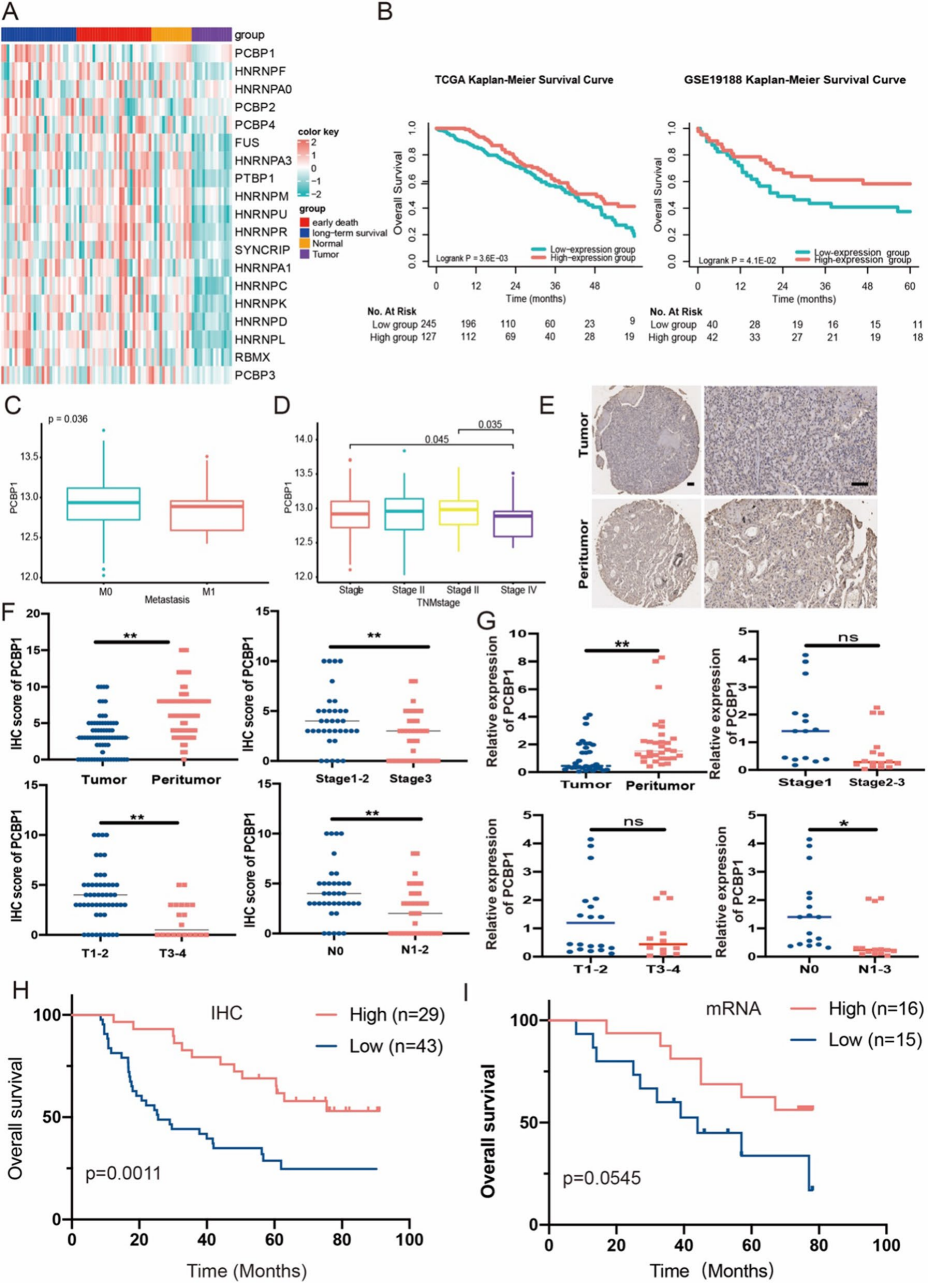
Identification of the prognostic and potential biological value of PCBP1. **A** Heatmap of differentially expressed genes between cancer and adjacent tissues in LUAD patients and the early death group and long-term survival group. **B** Kaplan–Meier survival curves of overall survival between high PCBP1 expression and low PCBP1 expression patients from TCGA and GSE19188. **C** PCBP1 expression between patients with distant metastasis and patients without metastasis. **D** PCBP1 expression in LUAD patients with different TNM stages. **E** IHC staining of human LUAD tissues and paired adjacent normal tissues; scale bars, 100 μm. **F**, **G** Comparison of PCBP1 detected by IHC staining (**F**) and qPCR (**G**) between tumour and peritumoral tissues and patients with different tumour sizes, N staging and tumour stages. **H** Kaplan–Meier analysis of overall survival was stratified by protein expression levels of PCBP1. **I** Kaplan–Meier analysis of overall survival was stratified by mRNA expression levels of PCBP1. *p < 0.05; **p < 0.01; ns, not significant

PCBPs are mostly involved in the regulation of gene expression at different levels. Some of these members play an important role in tumorigenesis. The hnRNP family was significantly downregulated in tumour tissues, while PCBP1 exhibited the most significant differential expression between the early death group and the long-term survival group (Fig. 1A). Expression of PCBP1 in lung tissue was relatively high (Additional file 2: Fig. S1A) and significantly correlated with the molecular or immune subtypes of LUAD (Additional file 2: Fig. S1B). Furthermore, the K-M survival curve showed that overall survival in the low PCBP1 expression group was significantly worse in both the TCGA and GSE19188 cohorts (Fig. 1B). The same results were also obtained in breast cancer, ovarian cancer, and gastric cancer cohorts from the K–M plotter database (Figure S1C). Furthermore, PCBP1 expression was significantly reduced in stage IV cancer tissues corresponding to patients with distant metastasis (Fig. 1C, D). PCBP1 also significantly correlated with the molecular or immune subtypes of pancancer patients (Additional file 2: Fig. S1D). These results suggest that PCBP1 plays an important role in lung tumorigenesis.

Next, we attempted to validate PCBP1 expression in LUAD and its predictive values for diagnosis. Immunohistochemistry showed that decreased expression of PCBP1 was observed in lung tumour tissues (Fig. 1E, F). There was a significant association between PCBP1 and tumour invasion, lymph node metastasis and clinical stage (Fig. 1F). We compared paired normal and tumour tissues of lung adenocarcinoma and found that PCBP1 mRNA levels were low in tumours (Fig. 1G). Moreover, we observed a more significant correlation between PCBP1 and the three pathological parameters at the mRNA level (Fig. 1G). Low expression of PCBP1 at both the protein and mRNA levels was closely correlated with poor survival in LUAD patients (Fig. 1H, I). Taken together, these data reveal that PCBP1 is a potential prognostic biomarker for LUAD in patients.

### PCBP1 inhibits proliferation and migration of LUAD cells in vitro

**Fig. 2:**
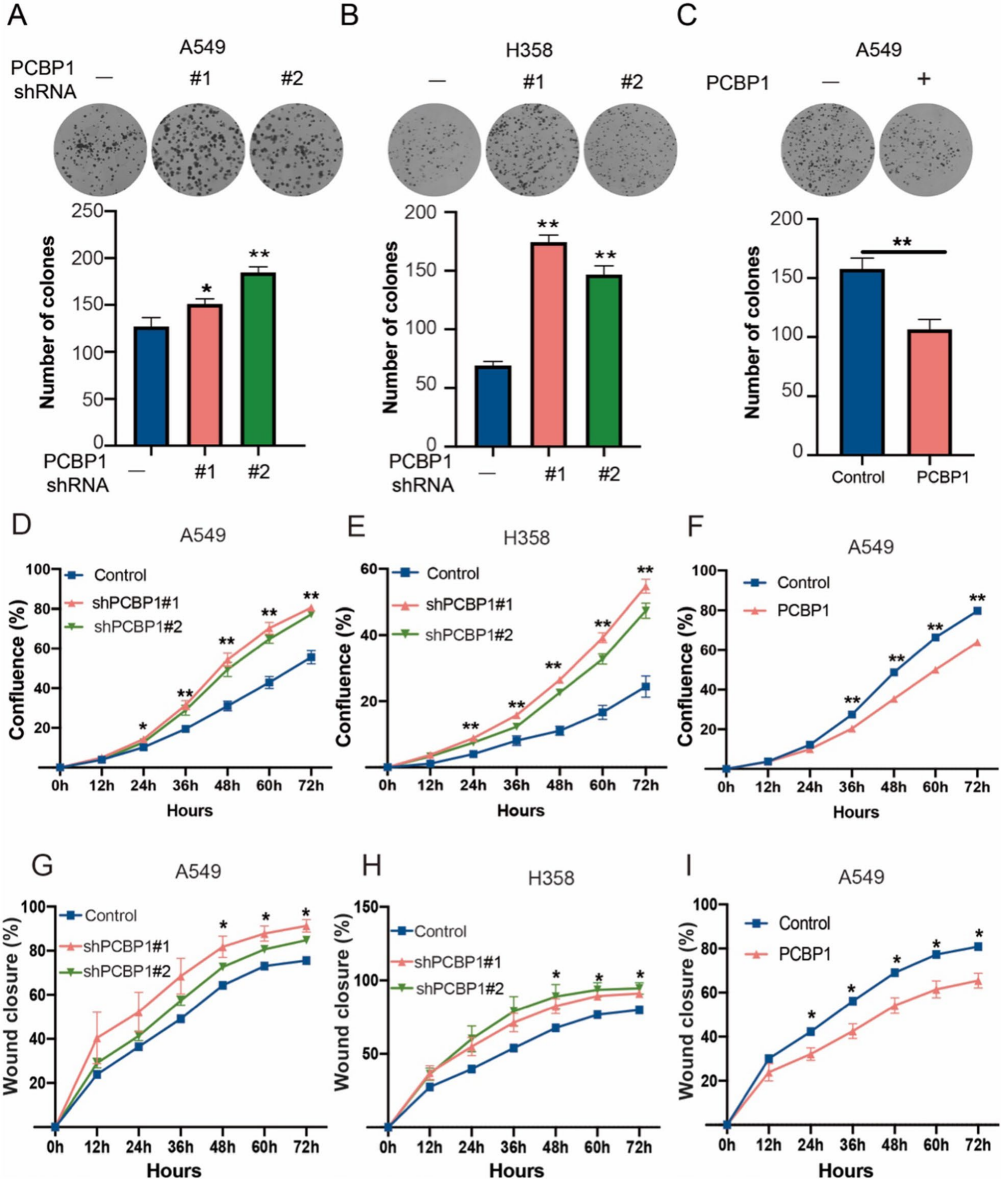
The effect of PCBP1 on the biological behaviour of LUAD in vitro. **A** shPCBP1 A549 cell colony formation ability. **B** shPCBP1 H358 cell colony formation ability. **C** PCBP1-OE A549 cell colony formation ability. **D**–**F** The proliferation rates of shPCBP1 A549 (**D**), shPCBP1 H358 (**E**), and PCBP1-OE A549 (**F**) cells were measured by IncuCyte ZOOM™. **G**–**I** Estimating the cell migration ability in shPCBP1 A549 (**G**), shPCBP1 H358 (**H**), and PCBP1-OE A549 (**I**) cells was performed by the IncuCyte™ Wound Healing assay. *p < 0.05; **p < 0.01; ns, not significant

We then generated PCBP1 stable knockdown cell lines using two short hairpin RNAs (shRNAs) in A549 and H358 cells. We confirmed the efficiency of PCBP1 deletion by western blot and qPCR (Additional file 2: Fig. S2A). We next explored the role of PCBP1 in LUAD progression. Colony formation and CCK-8 assays were performed to assess the effect of PCBP1 on cell proliferation. As shown, PCBP1 knockdown significantly increased colony formation (Fig. 2A, B) and cell proliferation (Additional file 2: Fig. S2B) in A549 and H358 cells. In contrast, PCBP1 overexpression decreased colony formation (Additional file 2: Fig. 2C) and cell proliferation (Additional file 2: Fig. S2C). Cell growth and wound healing can be dynamically observed using the IncuCyte™ assay. We observed that the cell proliferation rate in shPCBP1 cells was increased (Fig. 2D, E), and PCBP1 overexpression inhibited cell proliferation (Fig. 2F). Moreover, wound healing assays demonstrated that shPCBP1 cells exhibited increased cell migration ability compared to the control cells (Fig. 2G, H, and Additional file 2: Fig. S2D), while these indicators were decreased in cells overexpressing PCBP1 (Fig. 2I and Additional file 2: Fig. S2E). We found that PCBP1 inhibits the progression of lung adenocarcinoma.

### PCBP1 correlates with tumour metastasis in patients with lung adenocarcinoma

**Fig. 3:**
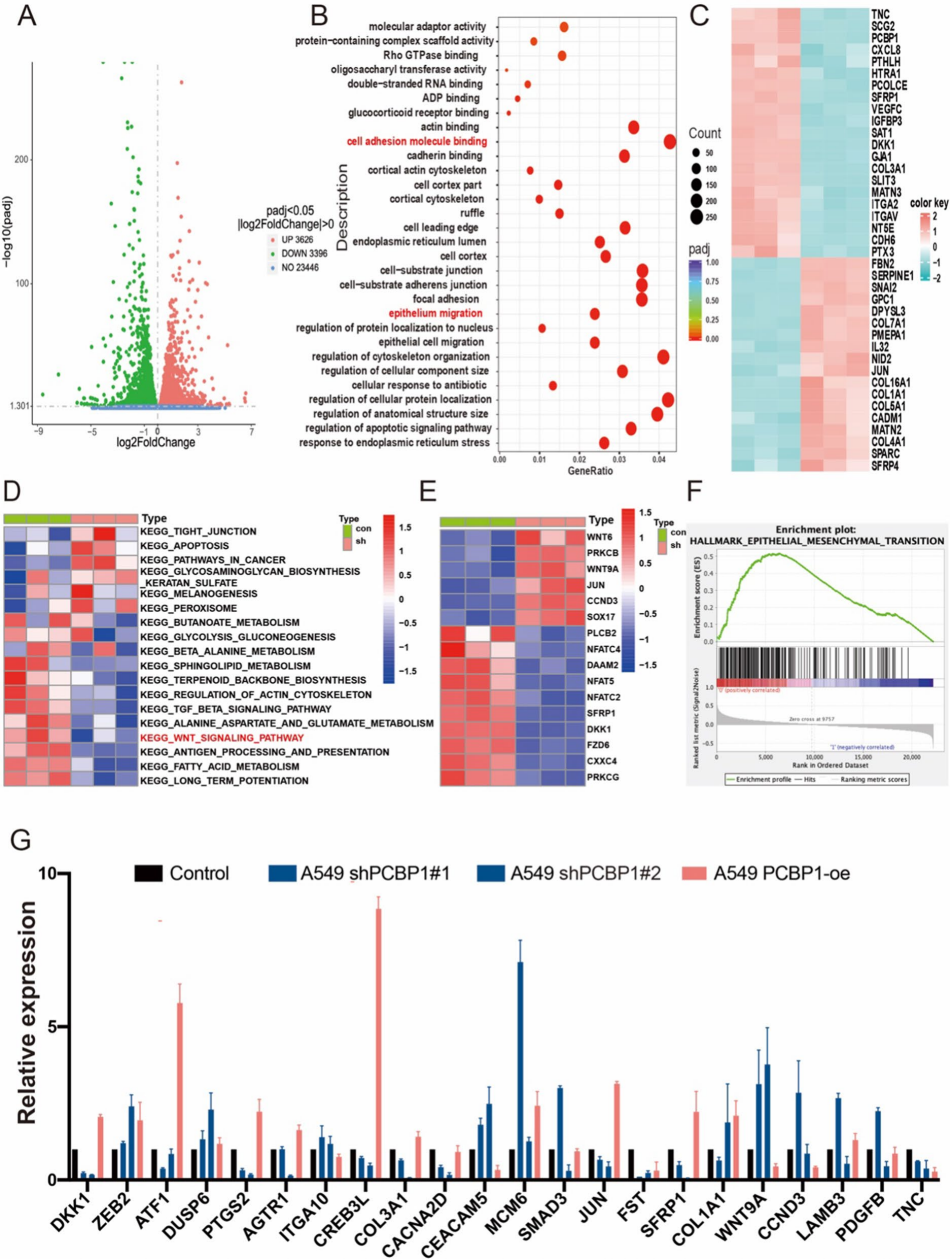
The expression of PCBP1 with respect to metastasis. **A** Volcano plot of DEGs in RNA-seq. **B** GO analysis showing the top gene functions that were mostly different among the PCBP1 high and low expression groups based on RNA-seq. **C** Heatmap showing the EMT-related DEGs identified by RNA-seq. **D** KEGG pathway enrichment analysis of DEGs. **E** The heatmap shows differential genes in WNT signalling pathways in A549 RNA‐seq data. **F** GSEA validated the enhanced activity of “Epithelial-Mesenchymal Transition”. **G** Validation of 22 selected genes by qPCR in PCBP1 knockdown and overexpression cells

To understand the molecular basis for PCBP1 on LUAD survival, we performed RNA-seq on A549 cells after PCBP1 depletion. We found large transcriptional changes, which included 643 downregulated genes and 490 upregulated genes (Fig. 3A). To further investigate the characteristics of the identified DEGs, we used gene oncology and KEGG pathway enrichment methods to analyse the intersecting DEGs. We observed that the DEGs were primarily enriched in biological processes associated with tumour metastasis: cell adhesion molecule binding, epithelium migration (Fig. 3B) and the Wnt signalling pathway (Fig. 3D). These pathways are all involved in tumour metastasis. Epithelial-mesenchymal transition (EMT) is an important biological process in tumour metastasis. EMT-related genes were acquired from the “HALLMARK_EPITHELIAL_ MESENCHYMAL_TRANSITION” gene list, which was displayed using a heatmap (Fig. 3C). GO pathway enrichment analysis of DEGs revealed that the enrichment pathway was primarily in metastasis-related signalling pathways, and in KEGG pathway analysis, the Wnt pathway regulated tumour metastasis. In the Wnt pathway, RNA-Seq analysis showed that 6 genes were upregulated and 10 genes were downregulated in PCBP1 knockdown cells compared to controls (Fig. 3E). According to the expression of PCBP1, LUAD patients in TCGA were divided into a high expression group and a low expression group. In the GSEA results, the “epithelial-mesenchymal transition” pathway was also enriched in the low PCBP1 expression group (Fig. 3F). We chose twenty-two DEGs based on fold-change and function to verify the RNA sequencing results by qRT–PCR in PCBP1 knockdown and overexpression cells (Fig. 3G). These findings revealed a link between PCBP1 and metastasis in LUAD patients.

### PCBP1 inhibits LUAD progression by directly binding to DKK1 mRNA and prolongs its mRNA stability

**Fig. 4:**
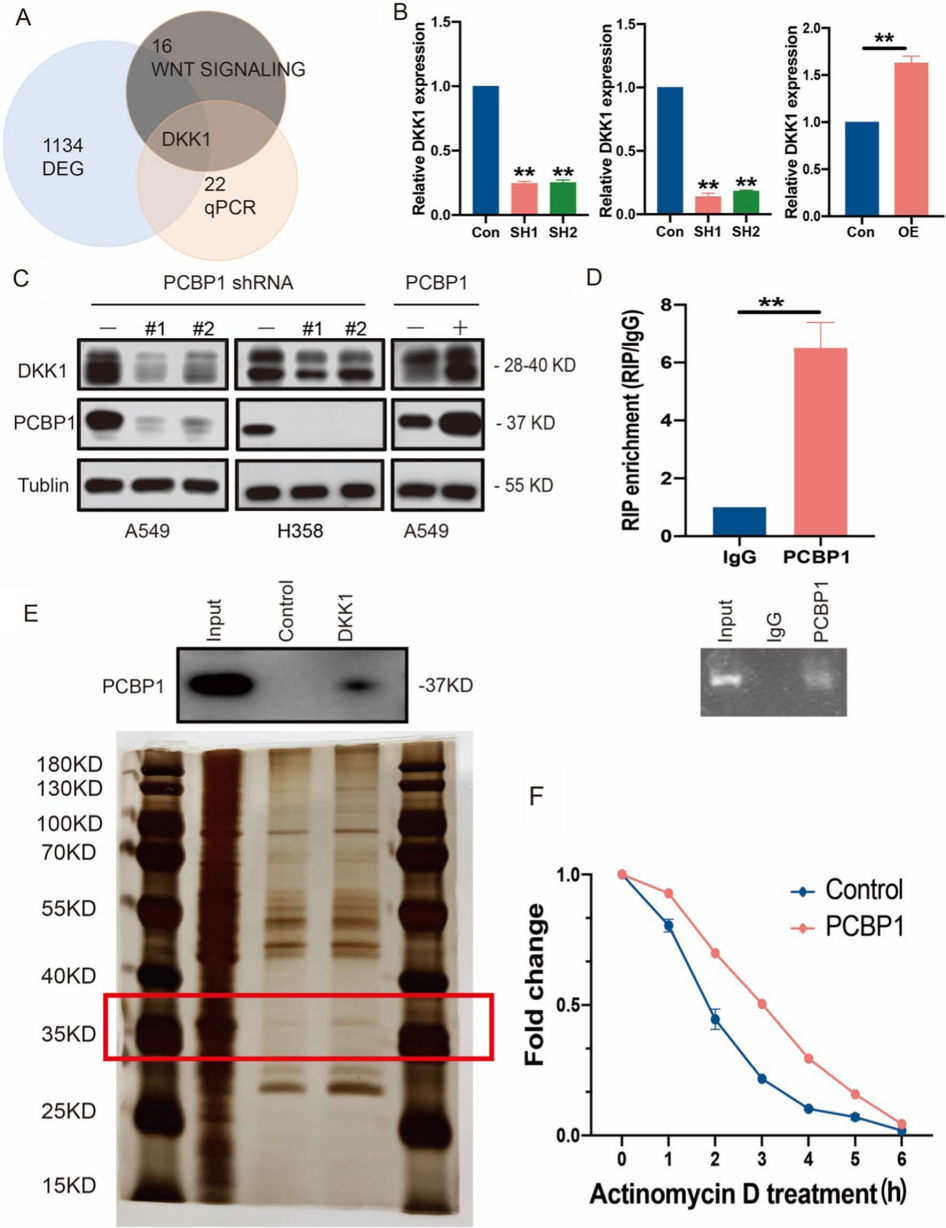
PCBP1 mediates DKK1 mRNA stability. **A** Graph based on the above results showing that DKK1 was the key gene. **B** qPCR analysis of the levels of DKK1 in PCBP1 knockdown and overexpression cells. **C** Western blot analysis of the levels of DKK1 in PCBP1 knockdown and overexpression cells. **D** The interaction of PCBP1 with DKK1 was examined by RIP-qPCR in A549 cells. **E** The interaction of PCBP1 with DKK1 was assessed by RNA pull-down assays followed by western blot analysis and silver staining. **F** mRNA expression of PCBP1-overexpressing cells and control cells treated with actinomycin D was examined by qPCR. *p < 0.05; **p < 0.01; ns, not significant

DKK1 belongs to the Wnt signalling pathway associated with lung cancer metastasis. Based on the above results, we found that DKK1 may be a key factor affecting tumour metastasis in response to PCBP1 alteration (Fig. 4A). The results indicated that PCBP1 knockdown in A549 and H358 cells decreased DKK1 protein levels, whereas overexpression of PCBP1 in A549 cells increased DKK1 mRNA levels (Fig. 4B). The same conclusions were observed at the protein level (Fig. 4C). PCBP1 has been identified as regulating alternative splicing, translation, and RNA stability of many cancer-related genes. To confirm the interaction between PCBP1 and DKK1, we performed RIP analysis. Surprisingly, RIP analysis revealed that DKK1 binds to PCBP1 in A549 cells (Fig. 4D). Moreover, RNA pulldown using A549 cell lysates followed by western blot analysis and silver staining (Fig. 4E) verified the combination of DKK1 with PCBP1 in the cells. We then assessed DKK1 mRNA stability in response to actinomycin D treatment. Indeed, overexpression of PCBP1 resulted in a prolonged half-life of DKK1 mRNA (Fig. 4F). These results demonstrate that PCBP1 closely governs DKK1 expression by stabilizing DKK1 mRNA.

### PCBP1 targets DKK1 to inhibit the Wnt/β-catenin pathway

**Fig. 5:**
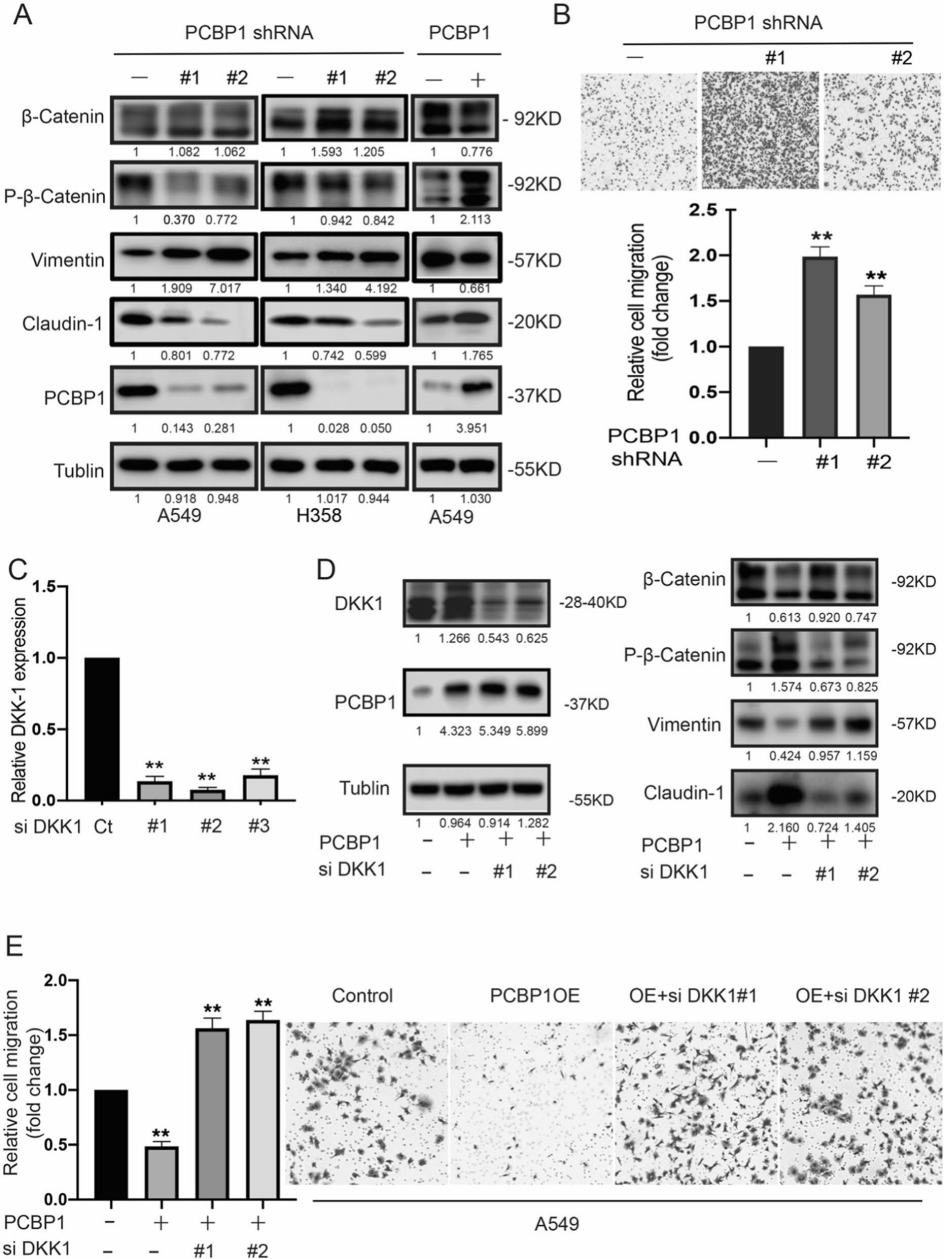
PCBP1/DKK1/β-catenin regulates migration and EMT in LUAD cells. **A** Western blotting analysis of the expression of β-catenin, phosphor-β-catenin, Vimentin, Claudin-1, PCBP1 and Tubulin. **B** shPCBP1 A549 cell migration was estimated by Transwell assays. **C** The knockdown efficiency of DKK1 in A549 cells was determined by RT–qPCR analyses. **D** Western blot assays were used to examine the expression levels of DKK1, β-catenin, phospho-β-catenin, Vimentin, Claudin-1, PCBP1 and Tubulin in A549 control, A549 PCBP1-OE, A549 PCBP1-OE + siDKK1#1, and A549 PCBP1-OE + siDKK1#2 cells. (E) Cell migration was determined by Transwell assay in each group. *p < 0.05; **p < 0.01; ns, not significant

DKK1 is an inhibitor of β‐catenin‐dependent Wnt signalling. Dysregulation of DKK1 has now emerged as an important factor in a variety of human cancers [35]. Western blot analysis revealed that β-catenin was upregulated in PCBP1 knockdown cells and downregulated in PCBP1-overexpressing cells. However, expression of phospho-β-catenin was the opposite (Fig. 5A). In addition, EMT-related marker levels were detected by western blot, indicating that PCBP1 reduced the levels of Vimentin but elevated claudin-1 levels (Fig. 5A). Transwell assays showed that shPCBP1 cells exhibited increased cell migration ability compared to control cells (Fig. 5B). We next examined whether PCBP1 suppresses cell migration and migration by regulating DKK1 in LUAD. We silenced DKK1 in A549 and PCBP1-OE A549 cells via siDKK1. The RT–qPCR results showed that si-DKK1#1 and si-DKK1#2 resulted in good knockdown efficiency (Fig. 5C). PCBP1-OE-transfected A549 cells presented elevated levels of phospho-β-catenin and claudin-1 and decreased levels of β-catenin and vimentin, but si-DKK1 reversed these results (Fig. 5D). We also explored the role of PCBP1 in cancer metastasis in LUAD. A549 cells with PCBP1 overexpression displayed markedly decreased cell migration ability, which was abrogated by downregulation of DKK1 (Fig. 5E). Collectively, these data demonstrate that DKK1 is an important downstream target of PCBP1.

### PCBP1 suppresses tumour growth and metastasis in LUAD in vivo

**Fig. 6:**
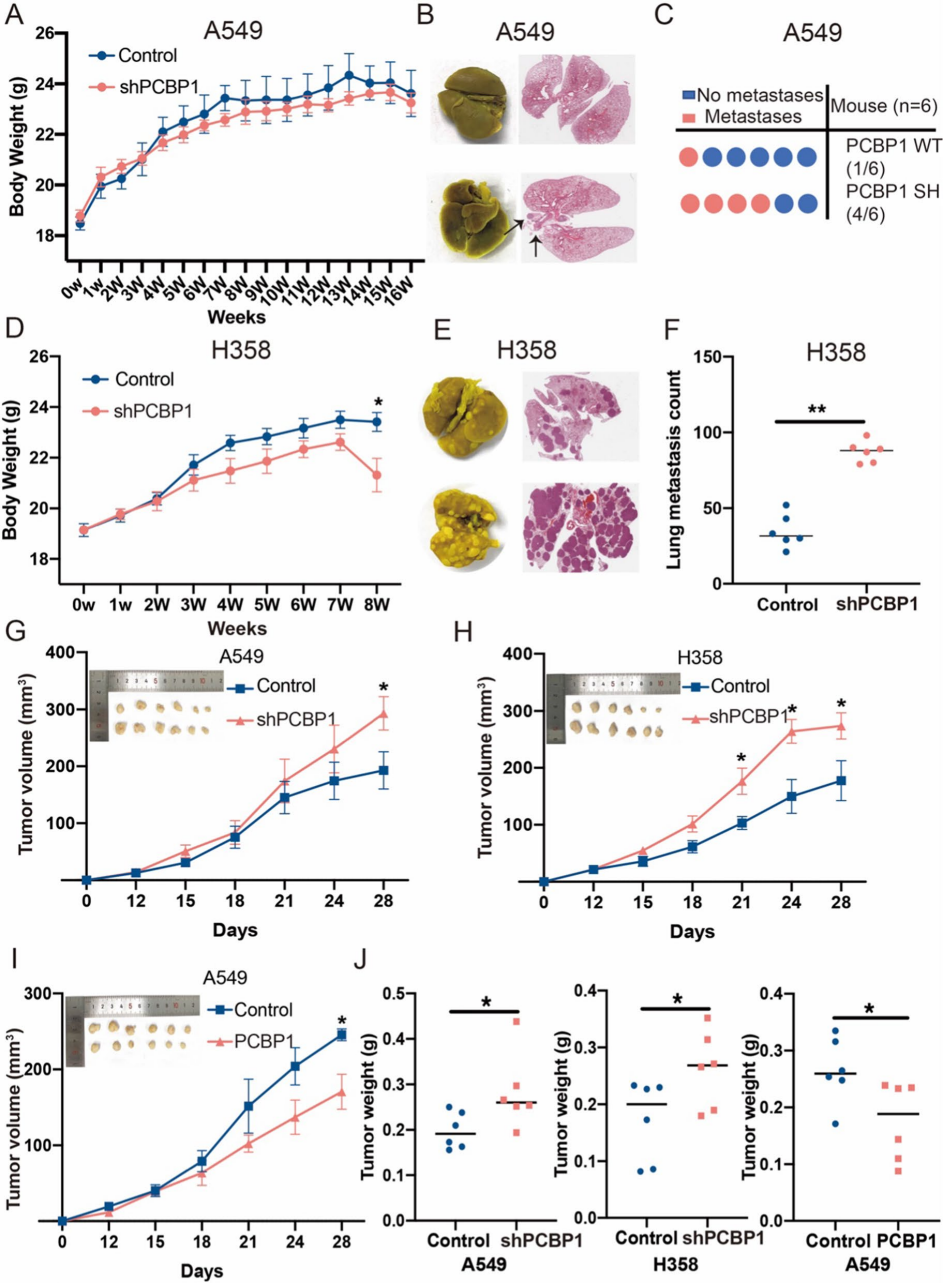
PCBP1 inhibits tumour growth and metastasis in LUAD in vivo. **A** Body weight of the mouse A549 cell lung metastasis model. **B** Representative macroscopic lung images upon necropsy of mice with postimplant shPCBP1 and control A549 cells and HE images. **C** Quantification of lung metastases in mice bearing either shPCBP1 or control tumours. **D** Body weight of the mouse H358 cell lung metastasis model. **E** Representative macroscopic lung images upon necropsy of mice with postimplant shPCBP1 and control H358 cells and HE images. **F** The number of nodules per lung was quantified by HE staining. **G**, **H** A549 and H358 cells stably transfected with control and shPCBP1 were injected subcutaneously into nude mice. Tumour growth curves were plotted. **I** A549 cells stably transfected with control and PCBP1 were injected subcutaneously into nude mice. Tumour growth curves were plotted. (J) The weights of the excised tumours were measured. *p < 0.05; **p < 0.01; ns, not significant

To explore the biological function of PCBP1 in lung metastasis, PCBP1 knockdown cells were injected into the tail vein of mice. PCBP1 knockdown significantly reduced body weight in H358 mouse models and exacerbated lung-metastatic burden both in A549 (Fig. 6A–C) and H358 (Fig. 6D–F) mouse models. We also examined the effect of PCBP1 on tumorigenicity in vivo. In the subcutaneous xenograft model, tumours appeared to have larger tumour volumes in the PCBP1 knockdown groups than in the control groups (Fig. 6G, H). Tumour growth was substantially inhibited in the PCBP1 overexpression group, as shown by the smaller tumour size and lighter tumour weight (Fig. 6I). Furthermore, IHC analysis indicated that Ki67-positive cells in tumor tissues were significantly increased after transfected with shPCBP1 and decreased after transfected with PCBP1 (Additional file 2: Fig. S3). The weights of the tumours excised from BALB/c nu mice were measured, and PCBP1 suppressed tumour growth and weight (Fig. 6J). Altogether, these data suggest that PCBP1 mediates lung metastasis and LUAD tumour growth.

### PCBP1 is positively correlated with DKK1 in LUAD patients

**Fig. 7:**
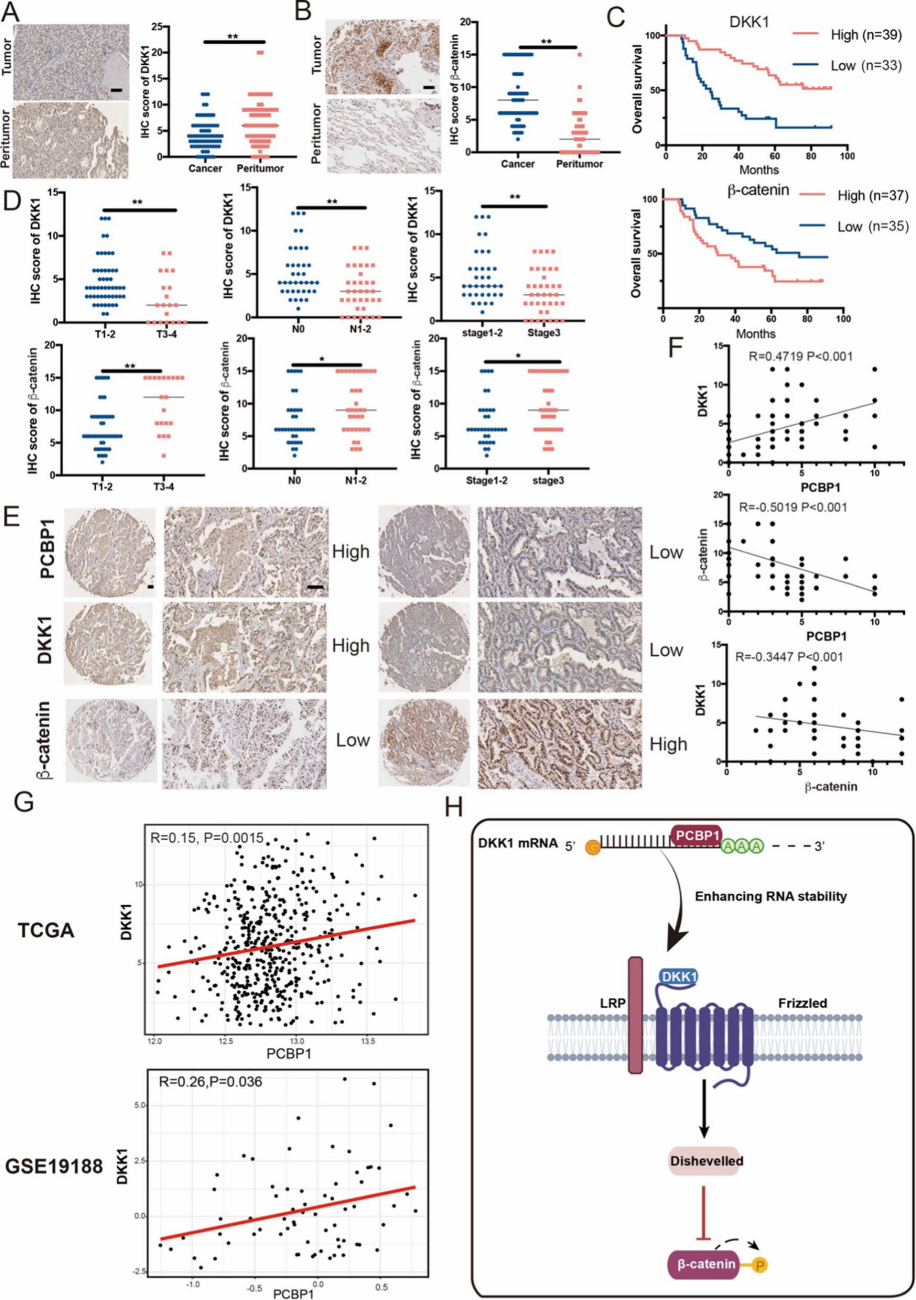
High levels of PCBP1 and DKK1 expression in LUAD predict good clinical outcome. **A** Representative images of DKK1 IHC staining; scale bars, 100 μm. The expression of DKK1 in peritumor and tumour tissues evaluated by immunohistochemistry. **B** Representative images of β-catenin IHC staining; scale bars, 100 μm. The expression of β-catenin in peritumor and tumour tissues evaluated by immunohistochemistry. **C** Kaplan–Meier analysis of overall survival was stratified by the protein expression levels of DKK1 and β-catenin. **D** Comparison of DKK1 and β-catenin protein expression detected by IHC between patients with different tumour sizes, N stages and tumour stages. **E** Representative images of PCBP1, DKK1 and β-catenin IHC staining; scale bars, 100 μm. **F** The correlation between PCBP1, DKK1and β-catenin at the protein level. **G** Correlation analysis of PCBP1 and DKK1 in TCGA and GSE19188. **H** Graphical summary of the PCBP1-DKK1- β-catenin axis regulating the proliferation and migration of lung cancer cells. *p < 0.05; **p < 0.01; ns, not significant

To examine the clinical relevance of these findings, we examined PCBP1, DKK1 and β-catenin expression in a clinically annotated cohort of 72 LUAD patients with paired adjacent normal tissues using immunohistochemistry staining. The data indicated that DKK1 protein expression was descended and β-catenin protein expression was enhanced in the tumor tissues compared to that in the paired normal lung tissues (Fig. 7A, B). High expression of DKK1 was closely correlated with improved survival in LUAD patients, while high expression of β-catenin predicts a poor prognosis (Fig. 7C). In parallel, DKK1 and β-catenin expressions detected using IHC staining were associated with tumor size, N staging and tumour stage (Fig. 7D). Tissues with a higher score for PCBP1 displayed increased DKK1 expression and decreased β-catenin. In contrast, tissues with a lower score for PCBP1 exhibited a lower level of DKK1 expression and higher level of β-catenin (Fig. 7E). These results indicate a positive correlation between PCBP1 and DKK1 in LUAD (Fig. 7F). And PCBP1 and DKK1 are both negatively correlated with β-catenin (Fig. 7F). The same results were verified in TCGA and GSE19188 databases (Fig. 7G). These findings demonstrate that PCBP1 is positively correlated with DKK1, and both PCBP1 and DKK1 are negatively correlated with β-catenin, which are independent prognostic markers of LUAD OS.

## Discussion

The hnRNP family contains many members involved in alternative splicing, mRNA stability, and translation regulation, and has been shown to play a regulatory role in a variety of diseases [36–38]. PCBPs is a subgroup belonging to the hnRNP family. PCBPs can be divided into two subgroups: hnRNP K/J and hnRNPE. HnRNP E protein consists of four members, namely PCBP1, PCBP2, PCBP3 and PCBP4. PCBPs has the common characteristics of three hnRNP K homology (KH) domains [39]. The amino acid sequences of PCBP 1 and PCBP 2 were highly similar (89%), but about 50% similar to those of the other three family members [40, 41]. However, according to current reports, the role of PCBP1 and PCBP2 in tumor is completely opposite, PCBP1 can be considered as a tumor suppressor, and PCBP2 plays a carcinogenic role. PCBP3 and PCBP4 have rarely been studied in cancer [38]. Through data analysis, we found that the expression of PCBP1 in patients with long survival time was higher than that in patients with short survival time, and the expression of PCBP1 in tumor tissues was significantly different from that in normal tissues. Therefore, we chose to functionally elucidate the role of PCBP1.

Loss of PCBP1 is implicated in various types of carcinogenesis [10, 13, 42]. Recently, PCBP1 has been shown to be a negative regulator of ovarian carcinoma [20], breast cancer [43], gastric cancer [22], and other cancers. EMT is a process in which epithelial cells acquire mesenchymal characteristics. In cancer, EMT is related to tumorigenesis, invasion, metastasis, and resistance to treatment [44]. Several recent studies have reported that PCBP1 is implicated in EMT markers [45, 46]. In addition, PCBP1 confers drug sensitivity in colorectal cancer, prostate cancer, and breast cancer [47]. All these findings highlight the clinical significance of PCBP1 in cancers. However, the exact role of PCBP1 in LUAD remains largely undefined.

In our study, we found that PCBP1 was decreased in LUAD tumour tissues compared to matched normal tissues. LUAD patients with high PCBP1 expression exhibited a longer survival time, and the data presented suggest that PCBP1 may act as a novel marker associated with good prognosis. Here, LUAD cells with silenced or overexpressed PCBP1 were established. Using colony formation assays, CCK8 assays, IncuCyte™ cell proliferation and wound healing and Transwell assays, we confirmed that PCBP1 was closely related to the proliferation and migration of LUAD cells. Mechanistically, the RNA-binding protein PCBP1 represses LUAD by directly binding to DKK1 mRNA. Downregulating expression of PCBP1 shortened the half-life of DKK1 mRNA and reduced its expression. Subsequently, the decreased expression of DKK1 protein downregulated beta-catenin phosphorylation, which relieves the inhibition of the Wnt/β-catenin signalling pathway. Disrupted β-catenin and other EMT markers (claudin-1 and vimentin) contribute to the malignant phenotype of LUAD cells. To fully elucidate the role of PCBP1 in LUAD, metastatic lung and subcutaneous xenograft models were successfully constructed. The data indicated that PCBP1 knockdown promoted lung metastasis and proliferation in vivo. Taken together, these results reveal that PCBP1 acts as a tumour suppressor gene, inhibiting the tumorigenesis of LUAD. Our findings highlight the potential of PCBP1 as a promising therapeutic target.

In addition to participating in tumour metastasis, PCBP1 has other important functions. PCBP1 is involved in regulating the stability of intracellular iron [48], and our study found that PCBP1 regulates ferroptosis in tumour cells (data not shown). PCBP1 also plays an important role in immune regulation. PCBP1 can promote the production of GM-CSF in T cells [49], PCBP1 is an intracellular immune checkpoint for shaping T-cell responses in cancer immunity [50], and PCBP1 modulates the innate immune response [51]. These studies suggest that PCBP1 may play a more complex role in tumours, which needs to be further investigated.

The tumorigenesis mechanisms of PCBP1 include execution of alternative splicing of oncogenes, inhibition of oncogene translation and regulation of mRNA stability [10]. PCBP1 is known to recognize poly(rC)- or CU-rich elements in its targets. It has been reported that PCBP1 can bind to the GCCCAG motif in the 5′-UTR of PRL-3 [42]. PCBP1 binds to the CU-rich motif in the 3′-UTR of P63 mRNA to stabilize p63 mRNA [16]. PCBP1 stabilizes sortilin mRNA by binding to atypical C-rich elements in the 3′-UTR [52]. PCBP1 also recognizes the atypical AU-rich element in the POLH mRNA 3′-UTR to stabilize POLH mRNA [53]. In this study, we found that PCBP1 directly binds to DKK1 to regulate the stability of DKK1 mRNA, but the specific binding region was not identified. Future studies are needed to clarify the specific binding elements or regions of PCBP1 and DKK1.

DKK1 is a secretory Wnt antagonist, and abnormal expression of DKK1 has become recognized as an important regulator of many human cancers. Some reports indicate that DKK1 is a tumour promoter in pancreatic cancer, oesophageal cancer, and hepatocellular carcinoma [54, 55]. In contrast, DKK1 expression is downregulated in thyroid cancer [56], cutaneous squamous cell carcinoma [57], and breast cancer [58], suggesting that DKK1 may have a tumour suppressive effect. The role of DKK1 in tumours is controversial and may play different roles in different organs and tumours [59]. The function of DKK1 expression in lung cancer progression and prognosis remains unclear. Knockdown of DKK1 sensitizes NSCLC cells to cisplatin [60]. However, it has also been reported that a decrease in DKK-1 in metastatic lung cancer cells eliminates microglial inhibition and increases the risk of metastasis [61]. We found that DKK1 inhibits LUAD development via degradation of β-catenin. These results may indicate that DKK1 may play different roles in different organs, cell populations, or tumour states. Extensive efforts have been made to block Wnt signalling using small molecules, and the most effective target in cancer is the complex between TCF and β-catenin because it modulates signalling at downstream nodes of the pathway, a goal that has proven difficult to achieve despite extensive efforts [62]. DKK1 significantly reduces the expression of β-catenin, and targeting PCBP1 reduces catenin to achieve the therapeutic effect of inhibiting the WNT signalling pathway. Therefore, by studying the regulatory mechanism of PCBP1, we provide a new approach for blocking the WNT signalling pathway through β-catenin, providing a new therapeutic perspective for LUAD patients.

## Conclusions

Overall, our data reveal a novel regulatory mechanism of PCBP1-DKK1-β-catenin in LUAD and demonstrate that PCBP1 may serve as a novel marker associated with good prognostic factors. Our findings highlight the potential of PCBP1 as a promising therapeutic target.

## Supplementary Information

**Additional file 1: Table S1.**Clinicopathological features of 72 patients with LUAD. **Table S2 **PCR primer sequences.

**Additional file 2: Figure S1.** PCBP1 expression affects prognosis and molecular or immune subtypes. (A) The expression of PCBP1 in normal tissue from GTEx databases. (B) Correlations between PCBP1 expression and immune subtypes across TCGA LUAD tumours. (C) Kaplan–Meier survival curves of patients with breast cancer, ovarian cancer, and gastric cancer with different PCBP1 expression. (D) Correlations between PCBP1 expression and molecular or immune subtypes across TCGA tumours, including breast invasive carcinoma, oesophageal carcinoma, liver hepatocellular carcinoma, lung squamous cell carcinoma, stomach adenocarcinoma, and uterine corpus endometrial carcinoma. **Figure S2.** PCBP1 inhibits the proliferation of lung cancer cells, related to Fig. 2. (A) Western blot and qPCR analysis confirming the effects of knocking down PCBP1 in A549 and H358 cells. (B) shPCBP1 A549 and H358 cell proliferation was analysed by CCK-8 assay. (C) PCBP1-OE A549 cell proliferation was analysed by CCK-8 assay. (D-E) wound healing image. *p < 0.05; **p < 0.01; ns, not significant. **Figure S3.** PCBP1 inhibits tumour growth in vivo. H&E staining and IHC staining assessed the level of Ki67 in tumor tissues

## Data Availability

Authors can confirm that all relevant data and materials are available on request from the authors.

## Abbreviations

PCBP1: PolyC-RNA-binding protein 1
LUAD: Lung adenocarcinoma
DKK1: Dickkopf-1
TCGA: The Cancer Genome Atlas
GEO: Gene Expression Omnibus
DEGs: Differentially expressed genes
EMT: Epithelial-mesenchymal transition
CCK-8: Cell Counting Kit-8
IHC: Immunohistochemistry
RIP: RNA immunoprecipitation
siRNA: Small interfering RNA
GAPDH: Glyceraldehyde 3-phosphate dehydrogenase
RNA-seq: RNA sequencing

## References

[1] Sung H, Ferlay J, Siegel RL, Laversanne M, Soerjomataram I, Jemal A, Bray F (2021). Global cancer statistics 2020: GLOBOCAN estimates of incidence and mortality worldwide for 36 cancers in 185 countries. CA Cancer J Clin.

[2] Dejima H, Hu X, Chen R, Zhang J, Fujimoto J, Parra ER, Haymaker C, Hubert SM, Duose D, Solis LM (2021). Immune evolution from preneoplasia to invasive lung adenocarcinomas and underlying molecular features. Nat Commun.

[3] Allemani C, Matsuda T, Di Carlo V, Harewood R, Matz M, Nikšić M, Bonaventure A, Valkov M, Johnson CJ, Estève J (2018). Global surveillance of trends in cancer survival 2000–14 (CONCORD-3): analysis of individual records for 37 513 025 patients diagnosed with one of 18 cancers from 322 population-based registries in 71 countries. Lancet.

[4] Dillekås H, Rogers MS, Straume O (2019). Are 90% of deaths from cancer caused by metastases?. Cancer Med.

[5] Xue B, Chuang CH, Prosser HM, Fuziwara CS, Chan C, Sahasrabudhe N, Kühn M, Wu Y, Chen J, Biton A (2021). miR-200 deficiency promotes lung cancer metastasis by activating Notch signaling in cancer-associated fibroblasts. Genes Dev.

[6] Chaffer CL, Weinberg RA (2011). A perspective on cancer cell metastasis. Science.

[7] Obenauf AC, Massagué J (2015). Surviving at a distance: organ-specific metastasis. Trends Cancer.

[8] Feng J, Li H, Li J, Meng P, Wang L, Liu C, Zhao S, Sun W, Zhang Y (2020). hnRNPK knockdown alleviates NLRP3 inflammasome priming by repressing FLIP expression in Raw264.7 macrophages. Redox Rep.

[9] Zhang X, Di C, Chen Y, Wang J, Su R, Huang G, Xu C, Chen X, Long F, Yang H, Zhang H (2020). Multilevel regulation and molecular mechanism of poly (rC)-binding protein 1 in cancer. Faseb J.

[10] Guo J, Jia R (2018). Splicing factor poly(rC)-binding protein 1 is a novel and distinctive tumor suppressor. J Cell Physiol.

[11] Zhang W, Shi H, Zhang M, Liu B, Mao S, Li L, Tong F, Liu G, Yang S, Wang H (2016). Poly C binding protein 1 represses autophagy through downregulation of LC3B to promote tumor cell apoptosis in starvation. Int J Biochem Cell Biol.

[12] Zhang W, Zhang S, Guan W, Huang Z, Kong J, Huang C, Wang H, Yang S (2020). Poly C binding protein 1 regulates p62/SQSTM1 mRNA stability and autophagic degradation to repress tumor progression. Front Genet.

[13] Zhang T, Huang XH, Dong L, Hu D, Ge C, Zhan YQ, Xu WX, Yu M, Li W, Wang X (2010). PCBP-1 regulates alternative splicing of the CD44 gene and inhibits invasion in human hepatoma cell line HepG2 cells. Mol Cancer.

[14] Giles KM, Daly JM, Beveridge DJ, Thomson AM, Voon DC, Furneaux HM, Jazayeri JA, Leedman PJ (2003). The 3′-untranslated region of p21WAF1 mRNA is a composite cis-acting sequence bound by RNA-binding proteins from breast cancer cells, including HuR and poly(C)-binding protein. J Biol Chem.

[15] Evans JR, Mitchell SA, Spriggs KA, Ostrowski J, Bomsztyk K, Ostarek D, Willis AE (2003). Members of the poly (rC) binding protein family stimulate the activity of the c-myc internal ribosome entry segment in vitro and in vivo. Oncogene.

[16] Cho SJ, Jung YS, Chen X (2013). Poly (C)-binding protein 1 regulates p63 expression through mRNA stability. PLoS ONE.

[17] Wagener R, Aukema SM, Schlesner M, Haake A, Burkhardt B, Claviez A, Drexler HG, Hummel M, Kreuz M, Loeffler M (2015). The PCBP1 gene encoding poly(rC) binding protein I is recurrently mutated in Burkitt lymphoma. Genes Chromosomes Cancer.

[18] Liu Y, Sethi NS, Hinoue T, Schneider BG, Cherniack AD, Sanchez-Vega F, Seoane JA, Farshidfar F, Bowlby R, Islam M (2018). Comparative molecular analysis of gastrointestinal adenocarcinomas. Cancer Cell.

[19] Chen Q, Gu M, Cai ZK, Zhao H, Sun SC, Liu C, Zhan M, Chen YB, Wang Z (2021). TGF-β1 promotes epithelial-to-mesenchymal transition and stemness of prostate cancer cells by inducing PCBP1 degradation and alternative splicing of CD44. Cell Mol Life Sci.

[20] Shi H, Li H, Yuan R, Guan W, Zhang X, Zhang S, Zhang W, Tong F, Li L, Song Z (2018). PCBP1 depletion promotes tumorigenesis through attenuation of p27(Kip1) mRNA stability and translation. J Exp Clin Cancer Res.

[21] Woosley AN, Dalton AC, Hussey GS, Howley BV, Mohanty BK, Grelet S, Dincman T, Bloos S, Olsen SK, Howe PH (2019). TGFβ promotes breast cancer stem cell self-renewal through an ILEI/LIFR signaling axis. Oncogene.

[22] Zhang ZZ, Shen ZY, Shen YY, Zhao EH, Wang M, Wang CJ, Cao H, Xu J (2015). HOTAIR long noncoding RNA promotes gastric cancer metastasis through suppression of poly r(C)-binding protein (PCBP) 1. Mol Cancer Ther.

[23] Ji FJ, Wu YY, An Z, Liu XS, Jiang JN, Chen FF, Fang XD (2017). Expression of both poly r(C) binding protein 1 (PCBP1) and miRNA-3978 is suppressed in peritoneal gastric cancer metastasis. Sci Rep.

[24] Jiang P, Li Z, Tian F, Li X, Yang J (2017). Fyn/heterogeneous nuclear ribonucleoprotein E1 signaling regulates pancreatic cancer metastasis by affecting the alternative splicing of integrin β1. Int J Oncol.

[25] Steeg PS (2016). Targeting metastasis. Nat Rev Cancer.

[26] Zhan T, Rindtorff N, Boutros M (2017). Wnt signaling in cancer. Oncogene.

[27] Zhong Z, Yu J, Virshup DM, Madan B (2020). Wnts and the hallmarks of cancer. Cancer Metastasis Rev.

[28] Yang J, Chen J, He J, Li J, Shi J, Cho WC, Liu X (2016). Wnt signaling as potential therapeutic target in lung cancer. Expert Opin Ther Targets.

[29] Nagy A, Munkacsy G, Gyorffy B (2021). Pancancer survival analysis of cancer hallmark genes. Sci Rep.

[30] Gyorffy B (2021). Survival analysis across the entire transcriptome identifies biomarkers with the highest prognostic power in breast cancer. Comput Struct Biotechnol J.

[31] Geuens T, Bouhy D, Timmerman V (2016). The hnRNP family: insights into their role in health and disease. Hum Genet.

[32] Schroder MS, Culhane AC, Quackenbush J, Haibe-Kains B (2011). survcomp: an R/Bioconductor package for performance assessment and comparison of survival models. Bioinformatics.

[33] Ru B, Wong CN, Tong Y, Zhong JY, Zhong SSW, Wu WC, Chu KC, Wong CY, Lau CY, Chen I (2019). TISIDB: an integrated repository portal for tumor-immune system interactions. Bioinformatics.

[34] Subramanian A, Tamayo P, Mootha VK, Mukherjee S, Ebert BL, Gillette MA, Paulovich A, Pomeroy SL, Golub TR, Lander ES, Mesirov JP (2005). Gene set enrichment analysis: a knowledge-based approach for interpreting genome-wide expression profiles. Proc Natl Acad Sci U S A.

[35] Huang Y, Liu L, Liu A (2018). Dickkopf-1: current knowledge and related diseases. Life Sci.

[36] Weiss IM, Liebhaber SA (1994). Erythroid cell-specific determinants of alpha-globin mRNA stability. Mol Cell Biol.

[37] Weiss IM, Liebhaber SA (1995). Erythroid cell-specific mRNA stability elements in the alpha 2-globin 3′ nontranslated region. Mol Cell Biol.

[38] Yuan C, Chen M, Cai X (2021). Advances in poly(rC)-binding protein 2: structure, molecular function, and roles in cancer. Biomed Pharmacother.

[39] Makeyev AV, Liebhaber SA (2002). The poly(C)-binding proteins: a multiplicity of functions and a search for mechanisms. RNA.

[40] Makeyev AV, Liebhaber SA (2000). Identification of two novel mammalian genes establishes a subfamily of KH-domain RNA-binding proteins. Genomics.

[41] Chkheidze AN, Liebhaber SA (2003). A novel set of nuclear localization signals determine distributions of the alphaCP RNA-binding proteins. Mol Cell Biol.

[42] Wang H, Vardy LA, Tan CP, Loo JM, Guo K, Li J, Lim SG, Zhou J, Chng WJ, Ng SB (2010). PCBP1 suppresses the translation of metastasis-associated PRL-3 phosphatase. Cancer Cell.

[43] Wang X, Guo J, Che X, Jia R (2019). PCBP1 inhibits the expression of oncogenic STAT3 isoform by targeting alternative splicing of STAT3 exon 23. Int J Biol Sci.

[44] Pastushenko I, Blanpain C (2019). EMT transition states during tumor progression and metastasis. Trends Cell Biol.

[45] Grelet S, Link LA, Howley B, Obellianne C, Palanisamy V, Gangaraju VK, Diehl JA, Howe PH (2017). A regulated PNUTS mRNA to lncRNA splice switch mediates EMT and tumour progression. Nat Cell Biol.

[46] Chaudhury A, Hussey GS, Ray PS, Jin G, Fox PL, Howe PH (2010). TGF-beta-mediated phosphorylation of hnRNP E1 induces EMT via transcript-selective translational induction of Dab2 and ILEI. Nat Cell Biol.

[47] Tripathi V, Shin JH, Stuelten CH, Zhang YE (2019). TGF-β-induced alternative splicing of TAK1 promotes EMT and drug resistance. Oncogene.

[48] Patel SJ, Frey AG, Palenchar DJ, Achar S, Bullough KZ, Vashisht A, Wohlschlegel JA, Philpott CC (2019). A PCBP1-BolA2 chaperone complex delivers iron for cytosolic [2Fe-2S] cluster assembly. Nat Chem Biol.

[49] Wang Z, Yin W, Zhu L, Li J, Yao Y, Chen F, Sun M, Zhang J, Shen N, Song Y, Chang X (2018). Iron drives T helper cell pathogenicity by promoting RNA-binding protein PCBP1-mediated proinflammatory cytokine production. Immunity.

[50] Ansa-Addo EA, Huang HC, Riesenberg B, Iamsawat S, Borucki D, Nelson MH, Nam JH, Chung D, Paulos CM, Liu B (2020). RNA binding protein PCBP1 is an intracellular immune checkpoint for shaping T cell responses in cancer immunity. Sci Adv.

[51] Liao CY, Lei CQ, Shu HB (2021). PCBP1 modulates the innate immune response by facilitating the binding of cGAS to DNA. Cell Mol Immunol.

[52] Yabe-Wada T, Matsuba S, Takeda K, Sato T, Suyama M, Ohkawa Y, Takai T, Shi H, Philpott CC, Nakamura A (2016). TLR signals posttranscriptionally regulate the cytokine trafficking mediator sortilin. Sci Rep.

[53] Ren C, Cho SJ, Jung YS, Chen X (2014). DNA polymerase η is regulated by poly(rC)-binding protein 1 via mRNA stability. Biochem J.

[54] Kimura H, Sada R, Takada N, Harada A, Doki Y, Eguchi H, Yamamoto H, Kikuchi A (2021). The Dickkopf1 and FOXM1 positive feedback loop promotes tumor growth in pancreatic and esophageal cancers. Oncogene.

[55] Niu J, Li W, Liang C, Wang X, Yao X, Yang RH, Zhang ZS, Liu HF, Liu FY, Pei SH (2020). EGF promotes DKK1 transcription in hepatocellular carcinoma by enhancing the phosphorylation and acetylation of histone H3. Sci Signal.

[56] Zhang W, Ruan X, Li Y, Zhi J, Hu L, Hou X, Shi X, Wang X, Wang J, Ma W (2022). KDM1A promotes thyroid cancer progression and maintains stemness through the Wnt/β-catenin signaling pathway. Theranostics.

[57] Yuan S, Zhang P, Wen L, Jia S, Wu Y, Zhang Z, Guan L, Yu Z, Zhao L (2021). miR-22 promotes stem cell traits via activating Wnt/β-catenin signaling in cutaneous squamous cell carcinoma. Oncogene.

[58] Lv C, Li F, Li X, Tian Y, Zhang Y, Sheng X, Song Y, Meng Q, Yuan S, Luan L (2017). MiR-31 promotes mammary stem cell expansion and breast tumorigenesis by suppressing Wnt signaling antagonists. Nat Commun.

[59] Zhuang X, Zhang H, Li X, Li X, Cong M, Peng F, Yu J, Zhang X, Yang Q, Hu G (2017). Differential effects on lung and bone metastasis of breast cancer by Wnt signalling inhibitor DKK1. Nat Cell Biol.

[60] Salim H, Zong D, Hååg P, Novak M, Mörk B, Lewensohn R, Lundholm L, Viktorsson K (2015). DKK1 is a potential novel mediator of cisplatin-refractoriness in non-small cell lung cancer cell lines. BMC Cancer.

[61] Gan DX, Wang YB, He MY, Chen ZY, Qin XX, Miao ZW, Chen YH, Li B (2020). Lung cancer cells-controlled Dkk-1 production in brain metastatic cascade drive microglia to acquire a pro-tumorigenic phenotype. Front Cell Dev Biol.

[62] Nusse R, Clevers H (2017). Wnt/β-catenin signaling, disease, and emerging therapeutic modalities. Cell.

