# Supplementary material for: The RNA-binding protein PCBP1 represses lung adenocarcinoma progression by stabilizing DKK1 mRNA and subsequently downregulating β-catenin
Source: J Transl Med. 2022 Jul 30;20:343. doi: 10.1186/s12967-022-03552-y (PMC9338556; doi:10.1186/s12967-022-03552-y)
Supplement: Supplementary file 1 — Additional file 1: Table S1.Clinicopathological features of 72 patients with LUAD. Table S2 PCR primer sequences. [file 12967_2022_3552_MOESM1_ESM.docx]

**Additional Table S1** Clinicopathological features of 72 patients with LUAD.

|  | **Clinical feature** | **N=72** |  |
| --- | --- | --- | --- |
|  | **Age（years）**  <60  ≥60  **Tumor isize**  ≤ 3 cm  >3 to ≤ 5 cm  >5 to ≤ 7 cm  >7 cm  **Lymph node metastasis**  Absent Present  **Pathological stage**  I, II III, IV | 33  39  12  40  18  2  37  35  35  37 |  |

**Additional Table S2** PCR primer sequences.

| Gene | Forward 5′- 3′ | Reverse 5′-3′ |
| --- | --- | --- |
| GAPDH | ACAACTTTGGTATCGTGGAAGG | GCCATCACGCCACAGTTTC |
| b-Actin | GAGATCACTGCCCTGGCACC | GATGGAGGGGCCGGACTCG |
| PCBP1 | CCTACTCGATTCAAGGACAACAC | GAGTTCATGGGTGGTTTGAGTAG |
| DKK1  WNT9A  ZEB2  ATF1  DUSP6  PTGS2  AGTR1  ITGA10  CREB3L  COL3A1  CACNA2D1  CEACM5  MCM6  SMAD3  FST  JUN  COL1A1  SFRP1  CCND3  LAMB3  PDGFB  TNC | CTCCGGTCATCAGACTGTGC  AGCAGCAAGTTCGTCAAGGAA  CAAGAGGCGCAAACAAGCC  AGGACTCATCCGACAGCATAG  GAAATGGCGATCAGCAAGACG  CTGGCGCTCAGCCATACAG  ATTTAGCACTGGCTGACTTATGC  AACATCACCCACGCCTATTCC  GCACCTGGACCACTTTACGG  GGAGCTGGCTACTTCTCGC  CCGTCACTATCAAATCATGGGTG  TCTTGGCTGATTGATGGGAAC  GAGGAACTGATTCGTCCTGAGA  TGGACGCAGGTTCTCCAAAC  ACGTGTGAGAACGTGGACTG  GAGCTGGAGCGCCTGATAAT  GAGGGCCAAGACGAAGACATC  TGACTTCAGGTCAAGGGATGGT  TACCCGCCATCCATGATCG  GCAGCCTCACAACTACTACAG  CTCGATCCGCTCCTTTGATGA  CCTTGCTGTAGAGGTCGTCA | CCGGCAAGACAGACCTTCTC  CCTTCACACCCACGAGGTTG  GGTTGGCAATACCGTCATCC  TTCTGCCCCGTGTATCTTCAG  CGACGACTCGTATAGCTCCTG  CGCACTTATACTGGTCAAATCCC  CAGCGGTATTCCATAGCTGTG  GTTGGTAGTCACCTAAGTGGC  AGCACAGGGTCATCAAAGAAG  GGGAACATCCTCCTTCAACAG  TGCTTGAACTTTCTCCGCTTC  CACTGGCTGAGTTATTGGCCT  CAAGGCCCGACACAGGTAAG  CCGGCTCGCAGTAGGTAAC  CACATTCATTGCGGTAGGTTTTC  CCCTCCTGCTCATCTGTCAC  CAGATCACGTCATCGCACAAC  ACATCGCTTGAGGATCTGGAA  AGGCAGTCCACTTCAGTGC  CCAGGTCTTACCGAAGTCTGA  CGTTGGTGCGGTCTATGAG  CCAACCTCAGACACGGCTA |
|  |  |  |
