# Supplementary material for: The RNA-binding protein PCBP1 represses lung adenocarcinoma progression by stabilizing DKK1 mRNA and subsequently downregulating β-catenin
Source: J Transl Med. 2022 Jul 30;20:343. doi: 10.1186/s12967-022-03552-y (PMC9338556; doi:10.1186/s12967-022-03552-y)
Supplement: Supplementary file 2 — Additional file 2: Figure S1. PCBP1 expression affects prognosis and molecular or immune subtypes. (A) The expression of PCBP1 in normal tissue from GTEx databases. (B) Correlations between PCBP1 expression and immune subtypes across TCGA LUAD tumours. (C) Kaplan–Meier survival curves of patients with breast cancer, ovarian cancer, and gastric cancer with different PCBP1 expression. (D) Correlations between PCBP1 expression and molecular or immune subtypes across TCGA tumours, including breast invasive carcinoma, oesophageal carcinoma, liver hepatocellular carcinoma, lung squamous cell carcinoma, stomach adenocarcinoma, and uterine corpus endometrial carcinoma. Figure S2. PCBP1 inhibits the proliferation of lung cancer cells, related to Fig. 2. (A) Western blot and qPCR analysis confirming the effects of knocking down PCBP1 in A549 and H358 cells. (B) shPCBP1 A549 and H358 cell proliferation was analysed by CCK-8 assay. (C) PCBP1-OE A549 cell proliferation was analysed by CCK-8 assay. (D-E) wound healing image. *p < 0.05; **p < 0.01; ns, not significant. Figure S3. PCBP1 inhibits tumour growth in vivo. H&E staining and IHC staining assessed the level of Ki67 in tumor tissues [file 12967_2022_3552_MOESM2_ESM.docx]

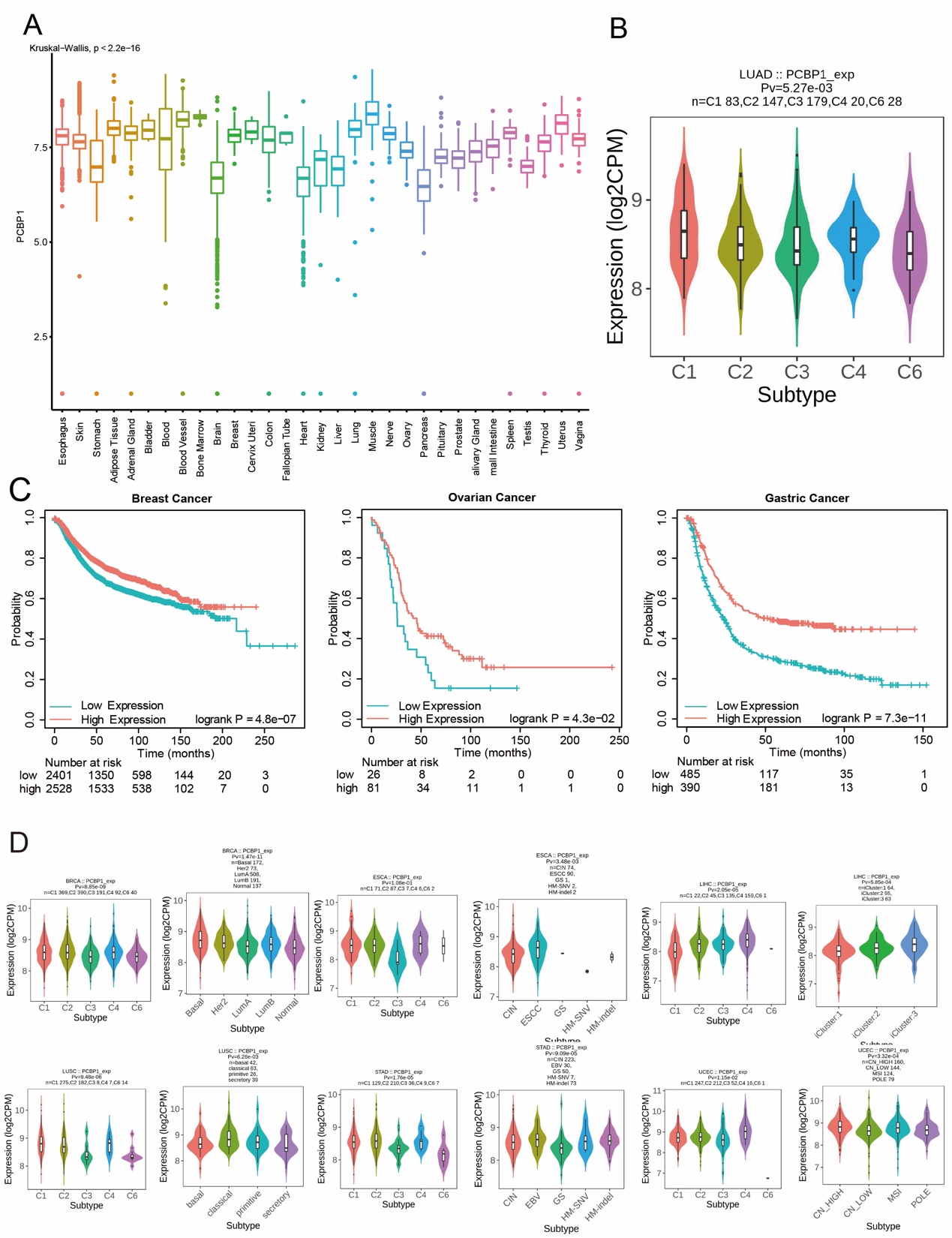


**Figure S1. PCBP1 expression affects prognosis and molecular or immune subtypes.**

(A) The expression of PCBP1 in normal tissue from GTEx databases. (B) Correlations between PCBP1 expression and immune subtypes across TCGA LUAD tumours. (C) Kaplan–Meier survival curves of patients with breast cancer, ovarian cancer, and gastric cancer with different PCBP1 expression. (D) Correlations between PCBP1 expression and molecular or immune subtypes across TCGA tumours, including breast invasive carcinoma, oesophageal carcinoma, liver hepatocellular carcinoma, lung squamous cell carcinoma, stomach adenocarcinoma, and uterine corpus endometrial carcinoma.


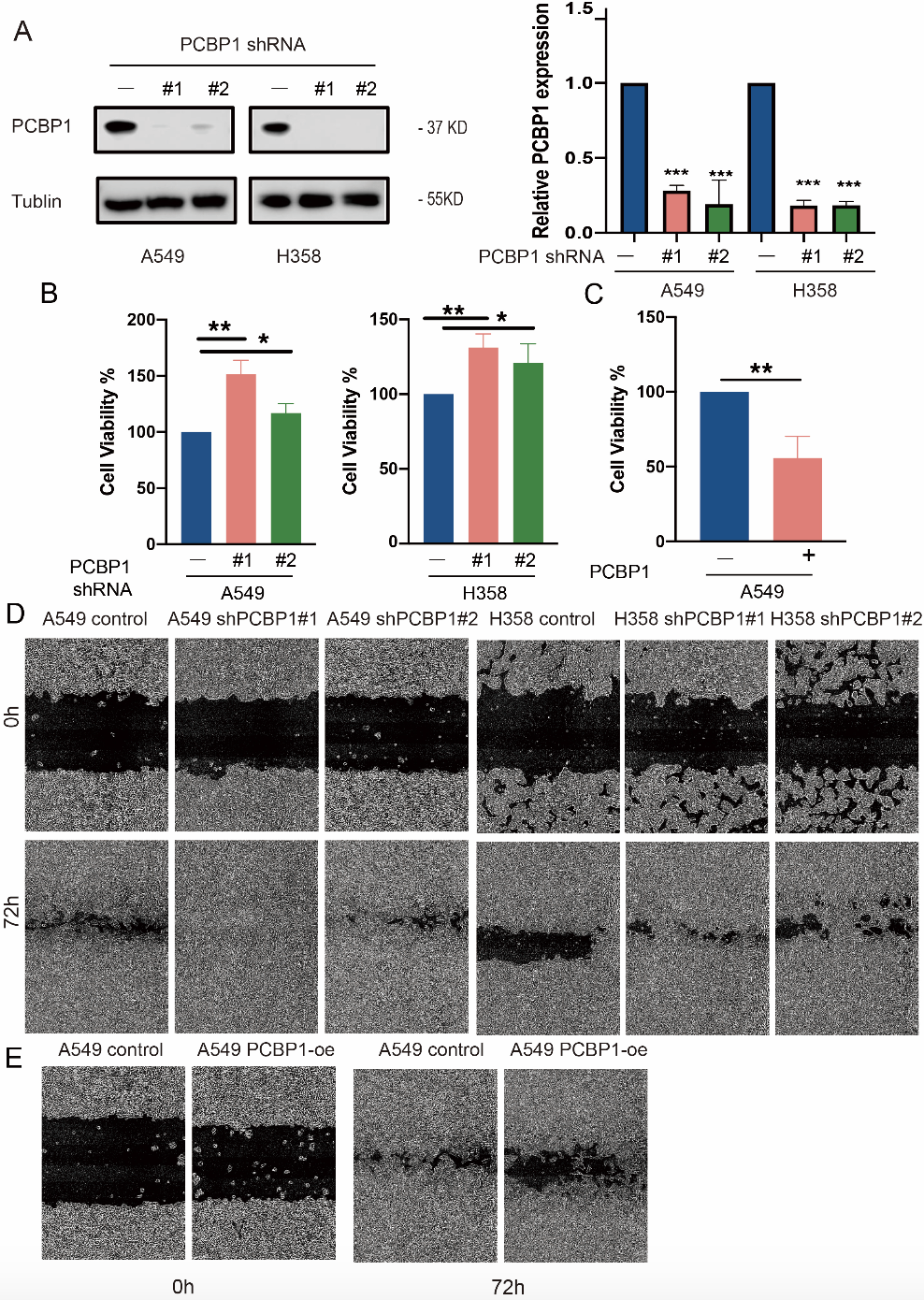


**Figure S2.** **PCBP1 inhibits the proliferation of lung cancer cells, related to Figure 2.**

(A) Western blot and qPCR analysis confirming the effects of knocking down PCBP1 in A549 and H358 cells. (B) shPCBP1 A549 and H358 cell proliferation was analysed by CCK-8 assay. (C) PCBP1-OE A549 cell proliferation was analysed by CCK-8 assay. (D-E) wound healing image. *p < 0.05; **p < 0.01; ns, not significant.


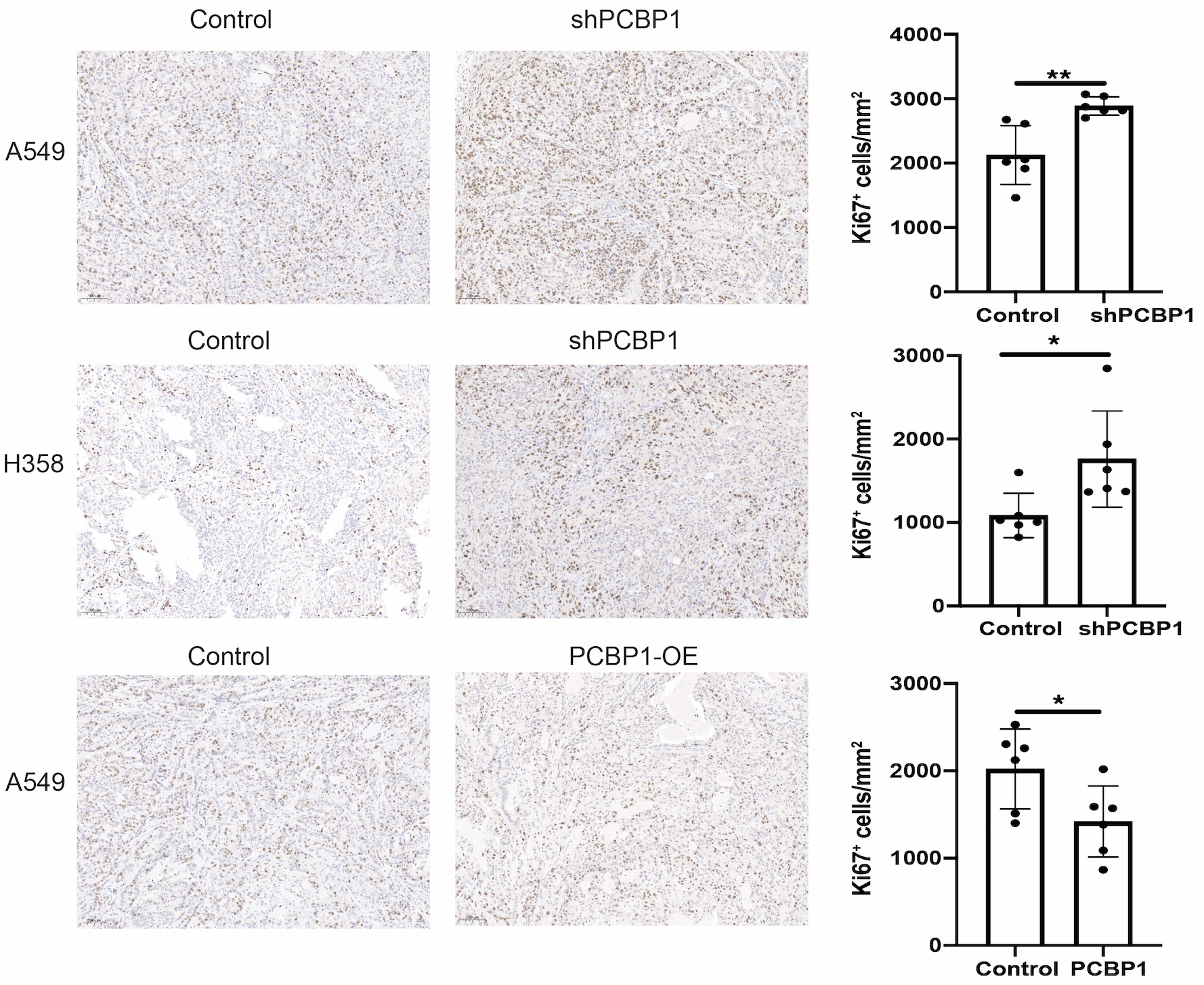
**Figure S3. PCBP1 inhibits tumour growth in vivo.**

H&E staining and IHC staining assessed the level of Ki67 in tumor tissues
